# Acupuncture for Rehabilitation After Total Knee Arthroplasty: A Systematic Review and Meta-Analysis of Randomized Controlled Trials

**DOI:** 10.3389/fmed.2020.602564

**Published:** 2021-01-18

**Authors:** Zehua Chen, Zhen Shen, Xiangling Ye, Yanfei Xu, Jinqing Liu, Xiaodong Shi, Guoqian Chen, Jiatao Wu, Weijian Chen, Tao Jiang, Wengang Liu, Xuemeng Xu

**Affiliations:** ^1^Kunming Municipal Hospital of Traditional Chinese Medicine, The Third Affiliated Hospital of Yunnan University of Chinese Medicine, Kunming, China; ^2^The Fifth Clinical Medical College of Guangzhou University of Chinese Medicine, Guangzhou, China; ^3^Guangdong Second Traditional Chinese Medicine Hospital, Guangzhou, China

**Keywords:** acupuncture, total knee arthroplasty, pain, function, systematic review

## Abstract

**Background:** There is an increasing interest in acupuncture for promoting post-operative rehabilitation, but the effectiveness of acupuncture for rehabilitation after total knee arthroplasty (TKA) remains controversial.

**Objective:** This study aims to investigate the effect of acupuncture on rehabilitation after TKA.

**Methods:** Database searches of PubMed, EMBASE, CINAHL, and China National Knowledge Infrastructure (CNKI) were conducted to obtain articles published until August 2020. All identified articles were screened, and data from each included study were extracted independently by two investigators. Meta-analysis was performed to examine the effects of acupuncture on pain, range of knee motion, function, and nausea/vomiting after TKA.

**Results:** A total of nine randomized clinical trials were included according to the inclusion and exclusion criteria in this review. Compared with routine treatment, acupuncture combined with routine treatment showed a significantly greater pain reduction at 8, 12, 24, and 48 h post-operatively after TKA. Meanwhile, we found that the acupuncture groups showed a significant function improvement and a lower percentage of nausea/vomiting in comparison with the control groups after operation. However, acupuncture groups demonstrated no statistically significant improvement in post-operative pain at 4 h, 7 days, 14 days, and more than 21 days, and no significant difference in range of knee motion was observed between the acupuncture groups and control groups after surgery.

**Conclusions:** Acupuncture, as a supplementary treatment after TKA, could improve function and reduce nausea/vomiting. However, the effect of acupuncture on pain relief may be mainly achieved within post-operative 48 h, and it had no efficacy in improving range of knee motion. More large-scale and high-quality studies are warranted.

## Introduction

Knee osteoarthritis (KOA) has become a major factor in disability for the elderly, which heavily compromises the quality of people's life (1). With the development of KOA, the destruction of articular cartilage will be deteriorated (2). For advanced KOA patients, total knee arthroplasty (TKA) is one of the common surgeries (3), which leads to a high degree of patient satisfaction because of considerable medium- and long-term benefits, including improvement in quality of life, pain relief, and function recovery (4). Nevertheless, TKA is followed by some complications, such as post-operative pain, limited joint function, and analgesia-related adverse effects, which exert a great impact on post-operative rehabilitation (5). Therefore, it is very important to take measures to manage the complications after TKA surgery, especially to explore a method to reduce post-operative pain (6). Historically, pharmacological methods, including anesthetics, opioids, and acetaminophen, serve as the first-line therapies to treat post-operative pain (7). However, drug-related adverse effects limit their application in daily clinical practice. Thus, many non-pharmacological approaches have developed, including preoperative exercise (8), cryotherapy (9), electrotherapy (10), and acupuncture (11), in order to obtain a better pain management and reduce the use of prescription medications.

Acupuncture, a traditional Chinese therapeutic technique with more than 3,000-year history, has been widely used to treat pain of many origins (12). Regarding the analgesic mechanism of acupuncture, it was suggested that needle stimulation to small-diameter nerve fibers could activate central nervous system at spinal level and higher brain center, and subsequently endogenous opioids and neurotransmitters would be generated and released (13). A previous meta-analysis indicated that certain modes of acupuncture could improve post-operative pain on the first day after surgery and reduced opioid use, and acupuncture should be considered an adjuvant therapy in treating post-operative pain (14). As was reported in another meta-analysis (15) including four randomized controlled trials (RCT), acupuncture was associated with reduced and delayed opioid consumption after TKA. In recent years, increasing number of RCTs investigated the efficacy of acupuncture in post-surgical recovery for patients receiving TKA. Some evidence (16–23) demonstrated that acupuncture could provide positive effects on pain management, reduction of swelling around the knee, early recovery range of motion (ROM), and hiccups for patients after TKA. On the contrary, two studies (24, 25) found that there was no significant effect on rehabilitation for patients with TKA. Consequently, clinical data examining the effectiveness of acupuncture for pain reduction and function improvement after TKA were inconsistent. The purpose of this systematic review and meta-analysis was to evaluate the effectiveness of acupuncture for rehabilitation after TKA.

## Methods

Ethical approval was not required in this review because all the analyses were based on data published in previous studies. This meta-analysis was carried out according to the Preferred Reporting Items for Systematic Review and Meta-analyses (26).

### Selection Criteria

In this study, the patients, interventions, comparators, and outcomes (PICO) question was taken into consideration at our primary search. All the included literatures should meet the following criteria: ① Study design: clinical randomized controlled study; ② Patients: patients undergoing primary TKA; ③ Intervention: acupuncture, including traditional acupuncture, needle, and dry needle; or acupuncture + other treatments; ④ Comparators: acupuncture vs. other treatments, acupuncture + other treatments vs. other treatments, acupuncture vs. placebo or sham acupuncture; ⑤ Outcomes: post-operative rehabilitation, at least one efficacy index; and ⑥ Languages: Chinese and English. Studies would be excluded if it met any of the following criteria: ① Conference abstracts, unpublished literatures, or full-text unavailable articles; ② Repeated publications, unicompartment knee arthroplasty, revision TKA, animal experimental studies, or reviews.

### Search Strategy

We searched electronic databases to obtain relevant studies, including PubMed, EMBASE, Cochrane Central Register of Controlled Trials (CINAHL), and China National Knowledge Infrastructure (CNKI) database, up to 29 August 2020. A search string such as “acupuncture,” “needle,” “total knee arthroplasty,” “total knee replacement,” “TKA,” “randomized controlled trial,” “controlled clinical trial,” “randomized,” “placebo,” “randomly,” and “trial,” etc. was used to search without restrictions. The search strategy is described in detail in Supplementary Figure 1. Two researchers (ZH Chen and JT Wu) performed preliminarily screening after careful reading of the title and abstracts. Subsequently, the remaining studies were screened strictly after reading the full text, and the eligible studies were included according to the inclusion and exclusion criteria. Finally, the data and materials in the included literatures were extracted without controversy. During the period of screening and data extraction, if discrepancies could not be resolved through discussion, the primary reviewer would be consulted.

### Data Extraction

The two investigators who screened the literatures independently extracted the main information from the included articles, including authors' names, publication year, age and gender of patients, study design, intervention type, acupuncture points, needle retaining time, intervention dose, main outcomes, and sample size.

### Quality Assessment

According to version 5.1.0 (27) of the Cochrane manual, the quality of the included literatures was qualitatively assessed with the risk of bias table by two reviewers (ZH Chen and WJ Chen). The literature was evaluated from seven aspects: sequence generation, allocation concealment, blind of participants and personnel, blind of outcome, incomplete outcome data, selective reporting, and other biases. The risk of each item is divided into three levels: high, unclear, and low.

### Statistical Analysis

We conducted the meta-system analysis of the observation indicators in the included literatures by using the review manager 5.3 software, and the results were illustrated by the forest map intuitively. The continuous variables were pooled by standard mean differences (SMDs) or mean differences (MDs) with 95% confidence intervals (95% CI), whereas the odds ratios (OR) were utilized to assess the enumeration data. Heterogeneity was assessed by the Cochran *Q* test and *I*^2^ index (28). An *I*^2^ statistic >50% was considered to be substantially heterogeneous. Based on the Cochrane Handbook for Systematic Reviews of Interventions (29), when the number of studies was <5 or studies are shown to be substantially heterogeneous, a random-effects model should be applied. Publication bias was assessed by the Begg's and Egger's tests. The difference was statistically significant when *P* < 0.05.

## Results

### Study Selection

**Figure 1:**
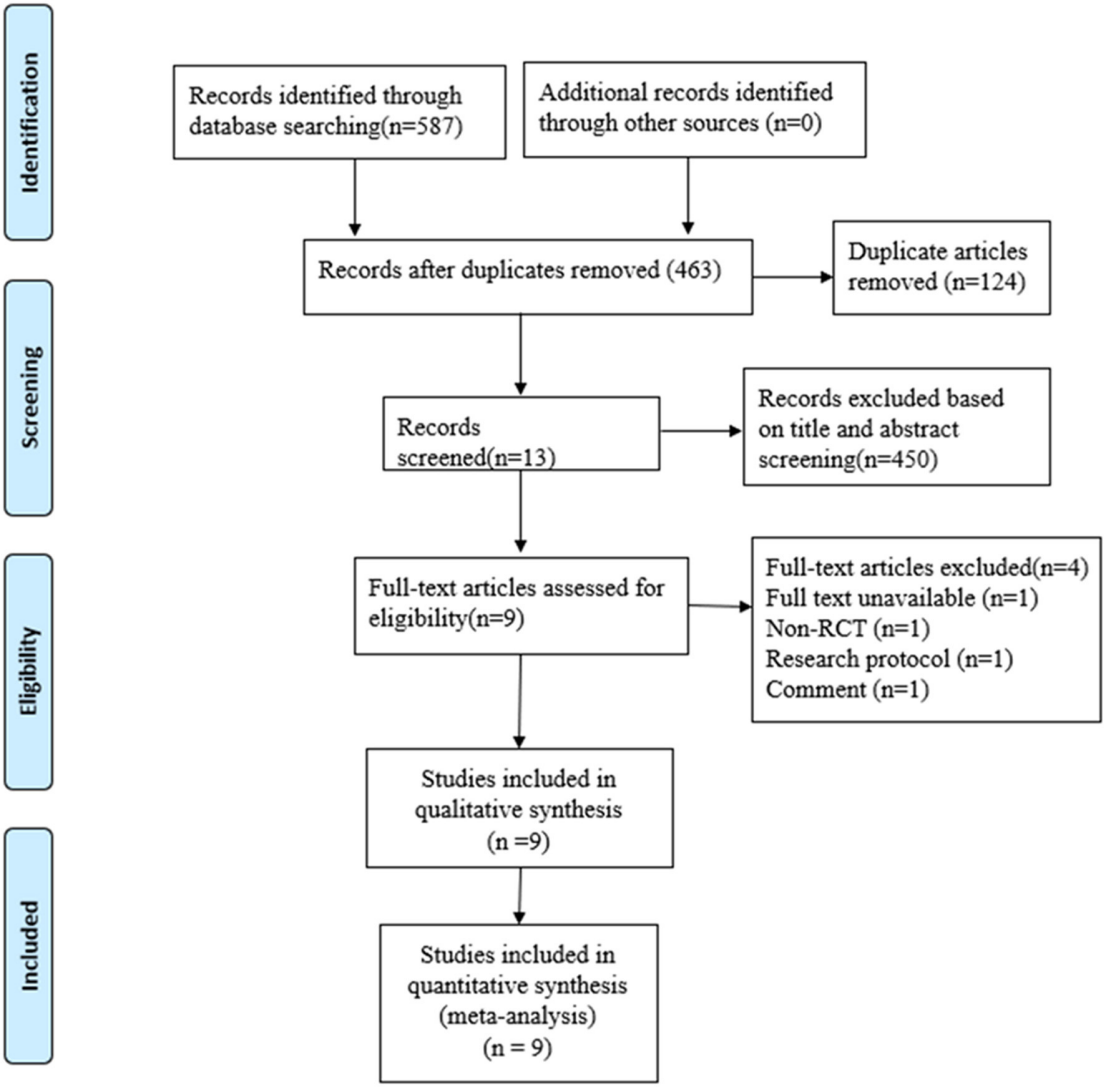
Flowchart of study selection.

A total of 587 studies were yielded by searching Chinese and English databases. After removing 124 duplicates and eliminating 454 articles through the preliminary screening, reading summary, and full text, 9 RCTs (18–22, 24, 25) were selected and 671 TKA patients were included. Four studies were excluded: one conference abstract, one comment, one without full text, and one observational study (Supplementary Table 1). The flowchart for the selection process is shown in Figure 1, and the basic information of each included literature is shown in Table 1. In this review, two (20, 25) of the included studies reported sham acupuncture applied in the control group (CG), and no acupuncture was used in the CG among the remaining studies. In the study reported by Tsang et al. (20), sham acupuncture was conducted as the needles were inserted <5 mm superficially and about 2 cm away from the same acupoints selected as in the acupuncture group (AG). Chen et al. (25) reported that auricular acupuncture needles for sham auricular acupuncture were specially treated, which could not be inserted into skin.

Table 1Characteristics of the included randomized clinical trials.

**Table:** 

**First author (y)**	**Country**	**Age(y)**		**Gender (female/male)**		**Sample size**	**Drop out**	**Study design**
		**AG**	**CG**	**AG**	**CG**	**(AG/CG)**	**(AG/CG)**	
Liao et al. (16)	China	70 ± 4.7	71.4 ± 3.2	11/19	13/17	30/30	0/0	RCT
Wang (17)	China	53.3 ± 4.5	54.1 ± 6.2	25/29	23/28	54/51	0/3	RCT
Zhang et al. (18)	China	67 ± 7	67 ± 9	14/16	13/17	30/30	0/0	RCT
Petersen et al. (24)	Denmark	56 ± 8	56 ± 6.8	49/38	52/33	87/85	3/2	RCT
Mikashima et al. (19)	Japan	72 ± 7	73 ± 5	10/30	12/28	40/40	0/0	RCT
Chen et al. (20)	Taiwan, China	68.9 ± 9.0	69.0 ± 8.6	3/27	8/22	31/31	1/1	RCT
Tsang et al. (25)	Hongkong, China	70.6 ± 5.8	66.1 ± 7.5	4/14	3/15	18/18	3/3	RCT
Mayoral et al. (21)	Spain	72.27 ± 6.95		9/11	2/18	20/20	3/6	RCT
Wang (22)	China	50~74		17/39		30/26	0/0	RCT

**Table:** 

**First author (y)**	**Intervention**		**Start time**		**Acupuncture points**	**Needle retaining time**	**Intervention Dose**
	**AG**	**CG**	**AG**	**CG**			
Liao et al. (16)	Analgesia drugs, routine rehabilitation, traditional manual acupuncture	Analgesia drugs, routine rehabilitation	From 2 d before surgery to post-operative 2 w		LI4 and LR3 in both sides	30 min	Once/d, 2w
Wang (17)	Analgesia pump, traditional manual acupuncture	Analgesia pump	2 d after operation		BL36, LR3, BL37, SP10, SP9, GB34	Not mentioned.	Once/d, 2w
Zhang et al. (18)	Analgesia pump, routine rehabilitation, traditional manual acupuncture	Analgesia pump, routine rehabilitation	1 d after operation		LR3, BL 60, KI3, BL 62, SP6 and SI3 in the operated side; LU5, LI11, and LI10 in both sides	30 min	Once/d, 2w
Petersen et al. (24)	Standard rehabilitation, western medical acupuncture	Standard rehabilitation	(2.2 ± 1.3) w after operation	(2.4 ± 1.6) w after operation	ST32, GB31, GB39, ST41, and LR3 in the operated side; SP10, ST34, LR8, SP9, and ST36 in the opposite side	15 20 min	Twice/w, 6w
Mikashima et al. (19)	Basic rehabilitation program, traditional manual acupuncture	Basic rehabilitation program	7 d after operation		ST31, 32, 38; SP6; BL-23, 25, 37, 57, 60; KI3; GB31,39, 40, 41, 42, and LR3 in the operated side	20 30 min	3 times/w, 2w
Chen et al. (20)	Auricular acupuncture and electroacupuncture	Only sham auricular acupuncture	Anesthetized and right before surgery started.		Auricular acupoints: TF4, AH4, AH6a and AT4; acupuncture points: SP10, ST34, BL40, LR7, ST36, GV20, and GV24	20 min	1session only
Tsang et al. (25)	Standard physiotherapy program and traditional manual acupuncture	Standard physiotherapy program and sham acupuncture	4 d after operation		ST32, ST33, GB31, GB35, GB34 and ST36	20 min	10 sessions within 2w
Mayoral et al. (21)	Dry needling	Sham dry needling	Anesthetized and right before surgery started.		Myofascial trigger points	Hong's fast-in, fast-out technique	1 session only
Wang (22)	Standard physiotherapy program and traditional manual acupuncture	Standard physiotherapy program.	3 d after operation		ST34, 35, 36, 41; SP10, SP9, GB33,34; LI4, BL40, EX-LE5	30 min	Twice/w, 6w

**Table:** 

**First author (y)**	**Time points for evaluation**	**Main outcome**	**Conclusion**	**Adverse events**
Liao et al. (16)	1, 2, 7, 14 d after operation	VAS, KSS	A significant improvement in pain and function in the AG	None
Wang (17)	14d after operation	VAS, HSS, ROM of knee	A significant improvement in pain and function in the AG	
Zhang et al. (18)	4, 8, 12, 24, 48 h, 7, 14 d after operation	VAS, HSS, ROM of knee, Analgesic consumption, Analgesia-Related Adverse Effects	A significant improvement in pain and ROM in the AG	None
Petersen et al. (24)	3 months of follow-up	KOOS pain, KOOS symptoms, KOOS-activities of daily living, KOOS quality of life, AROM-Ext, walking distance, No. with use of analgesics	No significant difference	None
Mikashima et al. (19)	7, 10, 13, 15, 17, 19, and 21 d after operation.	VAS, ratio of swelling around knee, Time to achieve preoperative range of motion	A significantly reduced pain and swelling around the knees and earlier recovery of ROM in the AG than those in the CG	None
Chen et al. (20)	At 2, 4, 8, 12, 24, 36, and 48 h after operation.	VAS, Analgesia-Related Adverse Effects, Analgesic consumption	A significant lower incidence of analgesia-related adverse effects of nausea and vomiting, and better pain control in the AG	None
Tsang et al. (25)	4–8 d and 11–15 d after operation	Numeric pain rating scale, Analgesics consumption, ROM of knee, Time up to go test	Acupuncture is not superior to sham acupuncture in pain relief and improvements of ranges of knee motion in patients undergoing bilateral total knee arthroplasty	2 patients refused to continue because of pain during acupuncture and 1 patient refused to continue because of pain during sham acupunctur
Mayoral et al. (21)	At 1, 3, 6 months after operation	VAS, WOMAC, ROM of knee, strength FLEX, and Strength EXT	A significant pain reduction in the first month after knee arthroplasty in the AG	None
Wang (22)	18 d after operation.	HSS	A significant improvement in function of knee in the AG	None

*AG, acupuncture group; CG, control group; VAS: AG: visual analog scale; ROM, range of motion; KOOS, Knee injury and Osteoarthritis and Outcome Score; HSS, Hospital for Special Surgery Knee Score; AROM-Ext, Active range of movement in knee extension (extension deficit); KSS, knee society score; TUG, time up to go test; RCT, randomized controlled trials*.

### Risk of Bias

**Figure 2:**
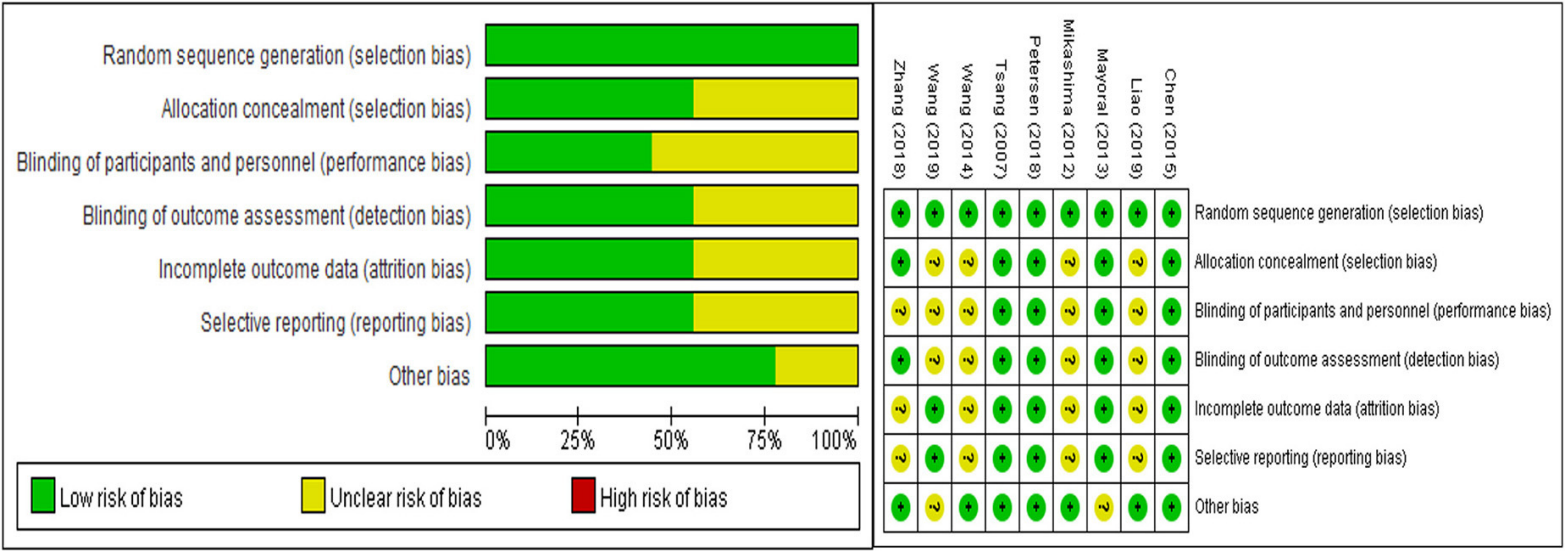
Risk of bias graph.

All the included studies were described as random generation, and five articles (18, 20–22, 24, 25) documented the methods of randomization in detail, in which a computer-generated random list was selected. Five of the 9 included studies recorded blind methods detailedly (20–22, 24, 25), and the dropout rate was reported in five articles (18, 20–22, 24, 25). The detailed results are shown in Figure 2.

### Meta-Analysis

#### Post-operative Pain

**Figure 3:**
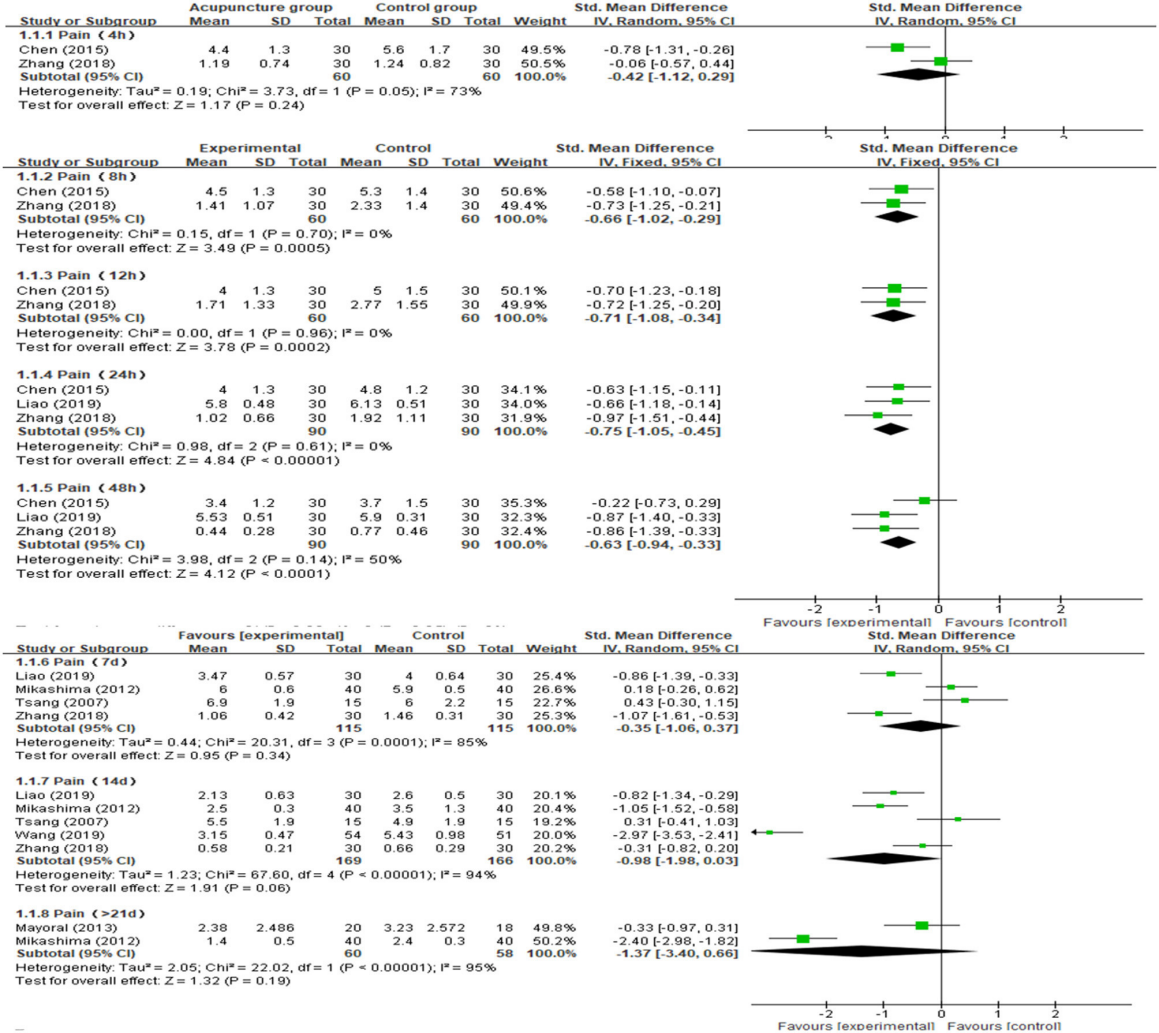
Meta-analysis and forest plot and for postoperative pain at different periods.

**Figure 4:**
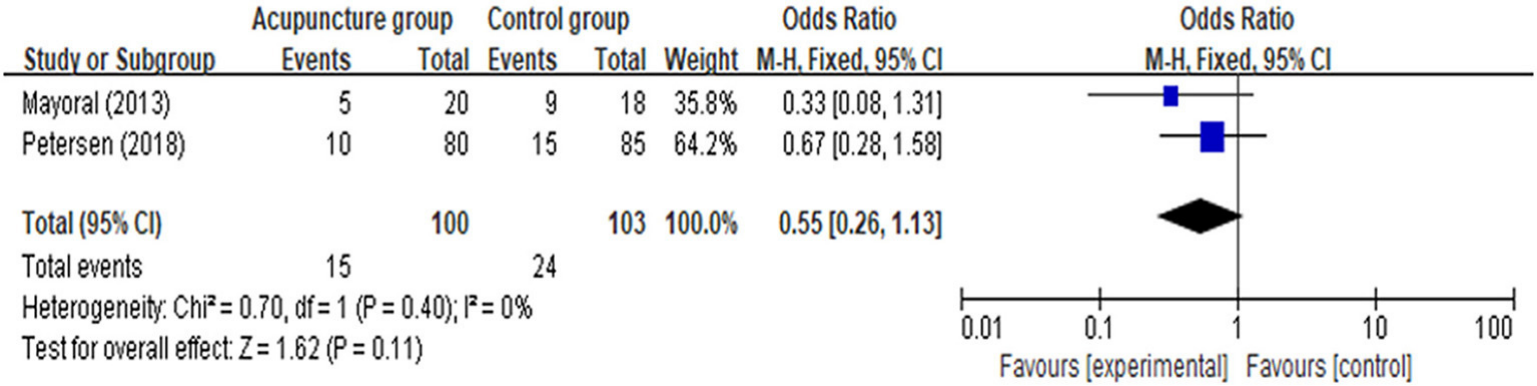
Meta-analysis and forest plot for number of patients with VAS score of more than 4.

**Figure 5:**
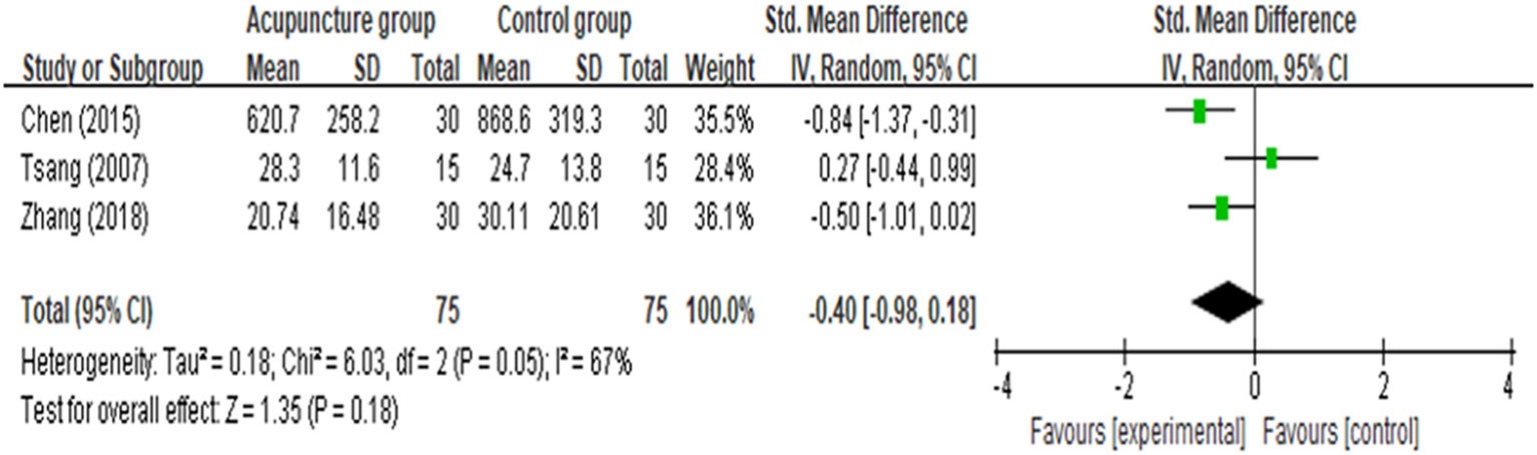
Meta-analysis and forest plot for analgesics consumption.

We preformed the meta-analyses to estimate the effects of acupuncture on pain at 4, 8, 12, 24, 48 h, 7, 14 days, and more than 21 days after TKA operation. From fixed-effects model, AG showed a significantly greater pain reduction compared with the CG at post-operative hour 8 [MD = −0.66 (95% CI: −1.02, −0.29), *p* = 0.0005, *I*^2^ = 0%], 12 [MD = −0.71 (95% CI: −1.08, −0.34), *p* = 0.0002, *I*^2^ = 0%], 24 [MD = −0.75 (95% CI: −1.05, −0.45), *p* < 0.00001, *I*^2^ = 0%], and 48 [MD = −0.63 (95% CI: −0.94, −0.33), *p* < 0.0001, *I*^2^ = 50%]. Analyses for the effect at the other time points in the post-operative period, including 4, 48 h, 7, 14 days, and more than 21 days, presented with substantial heterogeneity between the two groups (*I*^2^ = 73, 65, 87, 81, and 95%, respectively). Compared with CG, the results indicated AG had no statistically significant improvement in post-operative pain at 4 h [MD = −0.42 (95% CI: −1.12, 0.29), *p* = 0.24, *I*^2^ = 75%], 7 days [MD = −0.35 (95% CI: −1.06, 0.37), *p* = 0.34, *I*^2^ = 85%], 14 days [MD = −0.98 (95% CI: −1.98, 0.03), *p* = 0.06, *I*^2^ = 94%], and more than 21 days [MD = −1.37 (95% CI: −0.34, 0.66), *p* = 0.19, *I*^2^ = 95%] according to the random-effects model. Meta-analysis and forest plots are shown in Figure 3. Furthermore, there was no statistically significant difference in the number of patients with a score of more than 4 assessed by using VAS scale between the AG and the CG [OR = 0.55 (95% CI: 0.26, 1.13), *p* = 0.11, *I*^2^ = 0%; Figure 4]. In addition, no significant deference in post-operative analgesics consumption was observed between the both groups [MD = −0.14 (95% CI: −0.98, 0.18), *p* = 0.18, *I*^2^ = 67%; Figure 5].

#### ROM, Function, and Nausea/Vomiting (An Analgesia-Related Adverse Effect)

**Figure 6:**
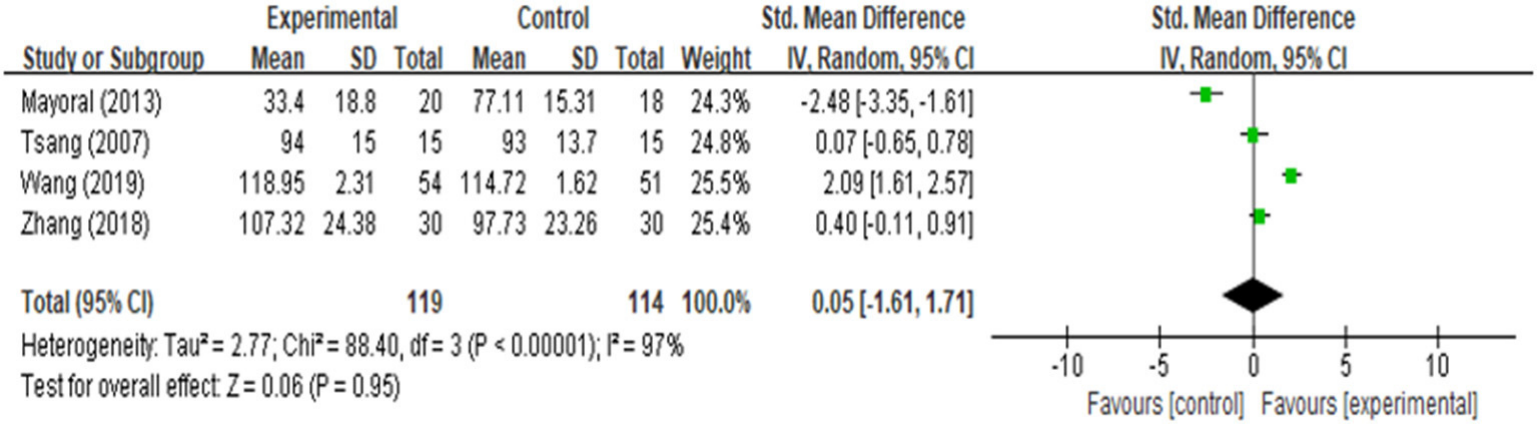
Meta-analysis and forest plot for ROM of knee.

**Figure 7:**
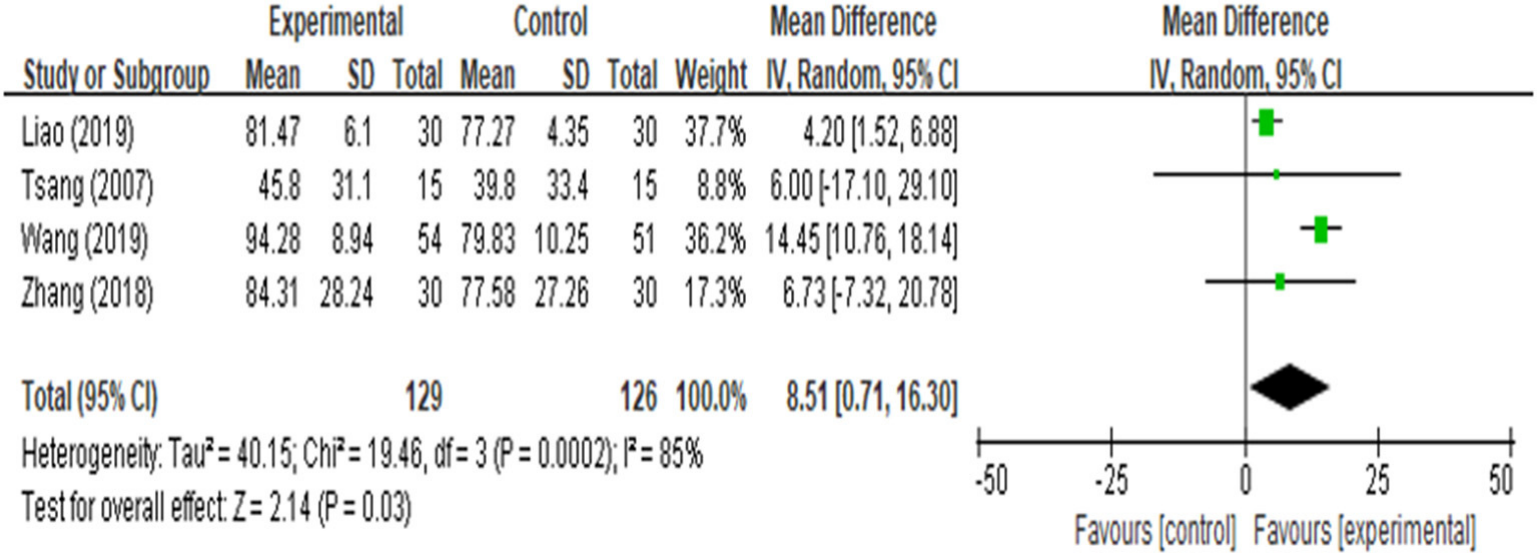
Meta-analysis and forest plot for function.

**Figure 8:**
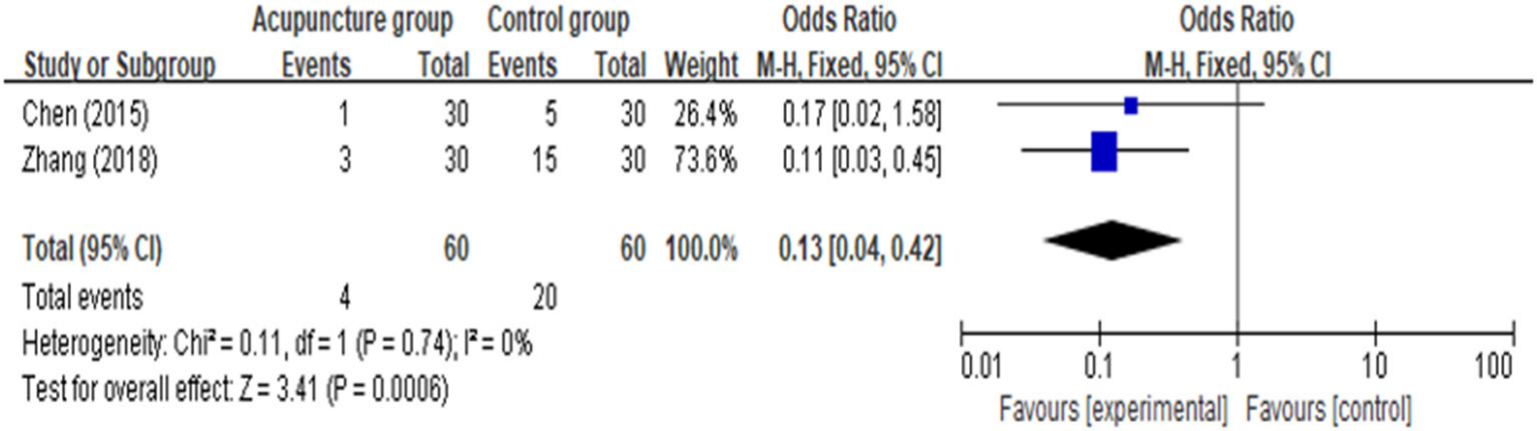
Meta-analysis and forest plot for nausea/vomiting.

ROM of knee was reported in four studies (17, 18, 21, 25), and the analysis result (Figure 6) revealed that it was closely similar in rehabilitation of knee ROM between AG and CG [MD = 0.05 (95% CI: −1.61, 1.71), *p* = 0.95, *I*^2^ = 97%]. Four of the 9 included studies documented function (16, 17, 22, 25), one of the three studies did not provide sufficient data, we attempted to contact authors but received no response. Four among the included studies reported function evaluated by using Hospital for Special Surgery Knee Score (HSS), either knee society score (KSS) or time up to go test (TUG). We found that there was a significant improvement in function [MD = 8.51 (95% CI: 0.71, 16.30), *p* = 0.03, *I*^2^ = 85%] between the AG and CG (Figure 7). However, meta-analysis of two studies (18, 20) showed AG had a significantly lower percentage of nausea/vomiting than the CG [OR = 0.13 (95% CI: 0.04, 0.42), *p* = 0.006, *I*^2^ = 0%; Figure 8].

#### Sensitivity Analysis

In this study, we conducted sensitivity analyses of knee ROM by excluding one study in each turn to detect the source of heterogeneity, while the overall heterogeneities and results were stable (Table 2). The analysis of function resulted in high heterogeneity due to the different assessment methods. Thus, the subgroup analysis was performed by the function evaluation, and we found that there was a significant improvement in the AG when compared with the CG [MD = 4.29 (95% CI: 1.66, 6.92), *p* = 0.001, *I*^2^ = 0%; Supplementary Figure 2].

**Table 2:** Sensitivity analysis for ROM of knee.

**Studies**	**Effect size**	**95% CI**	***P***	***I^**2**^ %***
Mayoral et al. (21)	0.87	−0.42, 2.15	0.18	94
Tsang et al. (25)	0.03	−2.19, 2.25	0.98	98
Wang (17)	−0.64	−2.24, 0.95	0.43	94
Zhang et al. (18)	−0.09	−2.65, 2.48	0.95	98

*ROM, range of motion; CI, confidence intervals*.

### Publication Bias

In this study, Begg's test and Egger's test were selected to examine publication bias, and no evidence for significant publication bias was found among the included studies. However, Egger's test for trials on post-operative pain at hours 4, 8, and 12 and >21 days, and nausea/vomiting were not available due to only two included trials for each test (Supplementary Table 2).

### Adverse Events

One of the included studies reported two patients in AG and one patient in CG refused to continue receiving acupuncture or sham acupuncture because of the intolerable pain.

## Discussion

Many previous studies (30–32) suggested that acupuncture was a potential method for pain relief, and it was widely used for the treatment and rehabilitation of acute or chronic pain. In order to achieve an adequate post-operative pain management, acupuncture was applied to relieve pain for patients undergoing TKA. Although one previous meta-analysis (12) including four RCTs had examined the efficacy of acupuncture in post-operative pain after TKA, pain at only two evaluation time points after operation, namely post-operative days 2 and 8, were analyzed. In the current study, we investigated the effects of acupuncture on pain after TKA at more evaluation time points, adding new evidence on acupuncture for pain reduction in patients receiving TKA. We found that patients treated with acupuncture and basic rehabilitation demonstrated a significant pain improvement when compared with patients receiving basic rehabilitation only at post-operative hours 8, 12, 24, and 48. However, acupuncture, as an adjunctive therapy, exhibited no significant superiority of improvement in post-operative pain at 4 h, 7, 14 days, and more than 21 days. In post-operative period, patients receiving continuous regional anesthesia showed a lower pain (33). For the pain assessment at post-operative hour 4, an undetectable difference in pain improvement between both groups might be found due to interferences of anesthesia resuscitation period. Overall, with regard to post-operative pain, these findings are in accordance with the results from the previous meta-analysis (12), indicating that pain relief benefits concentrate in the early post-operative phase but were ineffective in the long run. Moreover, the number of patients with moderate to severe pain (VAS score >4) in the two groups were balance, which revealed that acupuncture was no superior to decrease the ratio of patients with severe pain after TKA. In addition, no significant difference in analgesics consumption between AG and CG indirectly revealed the unobvious effect of acupuncture on pain reduction for TKA patients.

Postoperative pain could limit knee ROM; our study showed no evidence to support that acupuncture had any benefit in improving knee ROM for patients undergoing TKA. TKA was associated with post-operative pain, and most of TKA patients experience moderate to severe pain after surgery, which was detrimental to restore joint ROM. As mentioned above, acupuncture had no significant benefit in reducing pain for patients receiving TKA; hence, it was not helpful to enhance effectiveness for knee ROM. However, function represented a comprehensive performance, which could be influenced by many factors including proprioception, coordination, muscle strength etc., in addition to pain. Therefore, a significantly greater function improvement was examined in the AG than that in the CG at post-intervention.

The results of analgesia-related adverse effects are particularly notable. Analgesia drugs can cause numerous adverse effects such as nausea/vomiting, respiratory depression, hypotension, and pruritus (34). All the analgesia-related side effects could not be neglected because of its high prevalence of morbidity and seriousness which would result in decreased life quality and even significant mortality after major surgery (35). Among them, nausea/vomiting is the most common. To the best of our knowledge, this is the first meta-analysis to evaluate the efficacy of acupuncture on nausea/vomiting for TKA. The finding from our analysis was that acupuncture could significantly reduce the ratio of nausea/vomiting for patients undergoing TKA. The present finding is comparable with that reported in Becker's study (36), indicating that acupuncture as a complementary method could reduce nausea and vomiting after surgery.

Some limitations of this review should be considered. Firstly, most of the included studies did not record random and blind methods in detail. Secondly, the acupoints selected for treatment and intervention dose in the 9 included studies are not consistent, which may have led to overestimations or underestimations of the reported effects. Thirdly, because of the different assessment methods, a small number of studies was pooled in the analysis for the outcomes, which could contribute to a publish bias. However, according to the Begg's test and Egger's test, no evidence for significant publication bias was detected. Nevertheless, further trials with robust methodology are required to make more firm conclusions.

## Conclusion

In this work, we systematically reviewed and quantified the effects of acupuncture on post-operative rehabilitation for patients receiving TKA. Although acupuncture treatment exerted no positive effects on pain relief in the long stage and ROM of knee after TKA surgery, it achieved significant improvements in relieving the early post-operative pain and reducing the ratio of nausea/vomiting after TKA, which demonstrates that the use of acupuncture in the early post-operative period can be beneficial for the early post-operative rehabilitation. However, given the limitation in this study, additional high-quality and large-scale RCTs and systemic reviews are needed to confirm these findings.

## Data Availability Statement

The raw data supporting the conclusions of this article will be made available by the authors, without undue reservation.

## Author Contributions

ZC and ZS designed the study. ZC and ZS did the literature searches and designed the data-extraction form. YX, TJ, and ZC selected studies. XS, WC, and ZS extracted the data. GC, WL, XY, JL, and ZS did statistical analyses. ZC, JW, and XX supervised the study. ZS did the language editing. All authors read and approved the submitted version.

## Conflict of Interest

The authors declare that the research was conducted in the absence of any commercial or financial relationships that could be construed as a potential conflict of interest.
