# Supplementary material for: Acupuncture for Rehabilitation After Total Knee Arthroplasty: A Systematic Review and Meta-Analysis of Randomized Controlled Trials
Source: Front Med (Lausanne). 2021 Jan 18;7:602564. doi: 10.3389/fmed.2020.602564 (PMC7856874; doi:10.3389/fmed.2020.602564)
Supplement: Supplementary file 1 [file Table_3.DOCX]

Supplementary Material

**The search strategy used for Pubmed:**

| Query | Results |
| --- | --- |
| (((((((((((((((((((((((((((((((((Arthroplasties, Replacement, Knee) OR (Arthroplasty, Knee Replacement)) OR (Knee Replacement Arthroplasties)) OR (Knee Replacement Arthroplasty)) OR (Replacement Arthroplasties, Knee)) OR (Knee Arthroplasty, Total)) OR (Arthroplasty, Total Knee)) OR (Total Knee Arthroplasty)) OR (Replacement, Total Knee)) OR (Total Knee Replacement)) OR (Knee Replacement, Total)) OR (Knee Arthroplasty)) OR (Arthroplasty, Knee)) OR (Arthroplasties, Knee Replacement)) OR (Replacement Arthroplasty, Knee)) OR (Arthroplasty, Replacement, Partial Knee)) OR (Unicompartmental Knee Arthroplasty)) OR (Arthroplasty, Unicompartmental Knee)) OR (Knee Arthroplasty, Unicompartmental)) OR (Unicondylar Knee Arthroplasty)) OR (Arthroplasty, Unicondylar Knee)) OR (Knee Arthroplasty, Unicondylar)) OR (Partial Knee Arthroplasty)) OR (Arthroplasty, Partial Knee)) OR (Knee Arthroplasty, Partial)) OR (Unicondylar Knee Replacement)) OR (Knee Replacement, Unicondylar)) OR (Partial Knee Replacement)) OR (Knee Replacement, Partial)) OR (Unicompartmental Knee Replacement)) OR (Knee Replacement, Unicompartmental)) OR ("Arthroplasty, Replacement, Knee"[Mesh])) AND (((acupunctue[MeSH Terms]) OR (acupunctue)) OR (needle))) AND (randomized controlled trial[Publication Type] OR randomized[Title/Abstract] OR placebo[Title/Abstract]) | **42** |

**The search strategy used for EMBASE:**

#1 'knee arthroplasty'/exp **45727**

#2 'knee arthroplasty' **46158**

#3 'arthroplasty, replacement, knee' OR 'arthroplasties, replacement, knee' OR 'arthroplasty, knee replacement' OR 'knee replacement arthroplasties' OR 'knee replacement arthroplasty' OR 'replacement arthroplasties, knee' OR 'knee arthroplasty, total' OR 'arthroplasty, total knee' OR 'total knee arthroplasty' OR 'replacement, total knee' OR 'total knee replacement' OR 'knee replacement, total' OR 'knee arthroplasty' OR 'arthroplasty, knee' OR 'arthroplasties, knee replacement' OR 'replacement arthroplasty, knee' OR 'arthroplasty, replacement, partial knee' OR 'unicompartmental knee arthroplasty' **47105**

#4 'arthroplasty, unicompartmental knee' OR 'knee arthroplasty, unicompartmental' OR 'unicondylar knee arthroplasty' OR 'arthroplasty, unicondylar knee' OR 'knee arthroplasty, unicondylar' OR 'partial knee arthroplasty' OR 'arthroplasty, partial knee' OR 'knee arthroplasty, partial' OR 'unicondylar knee replacement' OR 'knee replacement, unicondylar' OR 'partial knee replacement' OR 'knee replacement, partial' OR 'unicompartmental knee replacement' OR 'knee replacement, unicompartmental'  **945**

#5 'acupuncture'/exp  **47318**

#6 'acupuncture' OR 'needle'#7 'randomized controlled trial'/exp **248702**

#7 #1 OR #2 OR #3 OR #4  **48697**

#8 #5 OR #6 **250986**

#9 #7 AND #8 **477**

#10 'randomized controlled trial'/exp OR 'controlled clinical trial'/exp OR 'randomized':ti,ab OR 'placebo':ti,ab OR 'drug therapy':lnk OR 'randomly':ti,ab OR 'trial':ti,ab OR 'groups':ti,ab  **7498450**

#11 #9 AND #10  **269**

**The search strategy used for CINAHL:**

#1 MeSH descriptor: [Arthroplasty, Replacement, Knee] explode all trees **2458**

#2 ((Arthroplasty, Replacement, Knee) OR (Arthroplasties, Replacement, Knee) OR (Arthroplasty, Knee Replacement) OR (Knee Replacement Arthroplasties) OR (Knee Replacement Arthroplasty) OR (Replacement Arthroplasties, Knee) OR (Knee Arthroplasty, Total) OR (Arthroplasty, Total Knee) OR (Total Knee Arthroplasty) OR (Replacement, Total Knee) OR (Total Knee Replacement) OR (Knee Replacement, Total) OR (Knee Arthroplasty) OR (Arthroplasty, Knee) OR (Arthroplasties, Knee Replacement) OR (Replacement Arthroplasty, Knee) OR (Arthroplasty, Replacement, Partial Knee) OR (Unicompartmental Knee Arthroplasty) OR (Arthroplasty, Unicompartmental Knee) OR (Knee Arthroplasty, Unicompartmental) OR (Unicondylar Knee Arthroplasty) OR (Arthroplasty, Unicondylar Knee) OR (Knee Arthroplasty, Unicondylar) OR (Partial Knee Arthroplasty) OR (Arthroplasty, Partial Knee) OR (Knee Arthroplasty, Partial) OR (Unicondylar Knee Replacement) OR (Knee Replacement, Unicondylar) OR (Partial Knee Replacement) OR (Knee Replacement, Partial) OR (Unicompartmental Knee Replacement) OR (Knee Replacement, Unicompartmental)):ti,ab,kw (Word variations have been searched) **7927**

#3 MeSH descriptor: [Acupuncture] explode all trees **150**

#4 ((acupuncture) OR (needle)):ti,ab,kw (Word variations have been searched) 27544

#5 #1 OR #2 **7927**

#6 #3 OR #4  **27544**

#7 #5 AND #6 **211**

**The search strategy used for CNKI:**

主题=针灸 or 主题= 针刺 or ( 题名= 针灸 or 题名=针刺) (精确匹配) and 主题=膝关节置换 or ( 题名= 膝关节置换) (精确匹配) **68**

**Supplementary eFigure 1** The search strategy in this review.


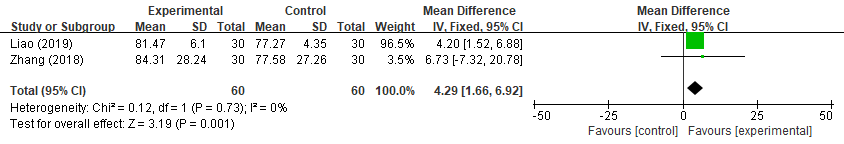


**Supplementary eFigure 2** Meta‐analysis and forest plot and for Hospital for Special Surgery Knee Score (HSS).

**Supplementary eTable 1** Excluded studies found from search strategy and reason.

| No | First author | Excluded randomised controlled trials | Reason |
| --- | --- | --- | --- |
| 1 | Y. Xiaolong | The effect of acupuncture during post-acute phase of rehabilitation after total knee arthroplasty | Conference Abstract |
| 2 | Xue FS | Acupuncture for Pain Relief After Total Knee Arthroplasty: A Call for Clarification | Comment study |
| 3 | R. Mast, | Acupuncture against postoperative pain after total knee replacement - A placebo-controlled trial on immediate effects | Full text  unavailable |
| 4 | Xu J | Treatment of persistent hiccups after arthroplasty: effects of acupuncture at PC6, CV12 and ST36 | Observation study |

**Supplementary eTable 2** Assessment of publication bias.

| Outcomes | N | Begg' s test | Egger's test |
| --- | --- | --- | --- |
| Postoperative pain (4 h) | 2 | 1 | NA |
| Postoperative pain (8 h) | 2 | 1 | NA |
| Postoperative pain (12 h) | 2 | 1 | NA |
| Postoperative pain (24 h) | 3 | 0.296 | 0.09 |
| Postoperative pain (48 h) | 3 | 0.296 | 0.065 |
| Postoperative pain (7 d) | 4 | 0.734 | 0.985 |
| Postoperative pain (14 d) | 5 | 0.806 | 0.745 |
| Postoperative pain (>21 d) | 2 | 1 | NA |
| Pain >40 | 2 | 1 | NA |
| ROM（14d） | 4 | 0.089 | 0.122 |
| Analgesics consumption | 3 | 1 | 0.297 |
| Function | 4 | 0.734 | 0.271 |
| Nausea/vomiting | 2 | 1 | NA |

N: number of studies; NA: not available.

1. Y. Xiaolong, W. Jianzhong. The effect of acupuncture during post-acute phase of rehabilitation after total knee arthroplasty. Annals of Physical and Rehabilitation Medicine. 2014, 57(1): e193.
2. Xue FS, Sun C, Li RP, et al. Acupuncture for Pain Relief After Total Knee Arthroplasty: A Call for Clarification. *Reg Anesth Pain Med*. 2015; 40(5): 640-641.
3. R. Mast, T. Schoch, H.-P. Scharf. Acupuncture against postoperative pain after total knee replacement - A placebo-controlled trial on immediate effects. *Aktuelle Rheumatologie*. 1995, 20(4): 131-134.
4. Xu J, Qu Y, Yue Y, et al. Treatment of persistent hiccups after arthroplasty: effects of acupuncture at PC6, CV12 and ST36. *Acupunct Med*. 2019; 37(1): 72-76.
